# Supplementary material for: A dynamic role for dopamine receptors in the control of mammalian spinal networks
Source: Sci Rep. 2020 Oct 2;10:16429. doi: 10.1038/s41598-020-73230-w (PMC7532218; doi:10.1038/s41598-020-73230-w)
Supplement: Supplementary file 1 — Supplementary Figures. [file 41598_2020_73230_MOESM1_ESM.pdf]

A dynamic role for dopamine receptors in the control of mammalian spinal networks

Simon A. Sharples[1], Nicole E. Burma [2,3], Joanna Borowska-Fielding[4], Charlie H.T. Kwok[2,5], Shane E.A. Eaton[2,5], Glen B. Baker[6], Celine Jean-Xavier[5], Ying Zhang[4], Tuan Trang[2,5], Patrick J. Whelan[2,5]

Supplementary Figures

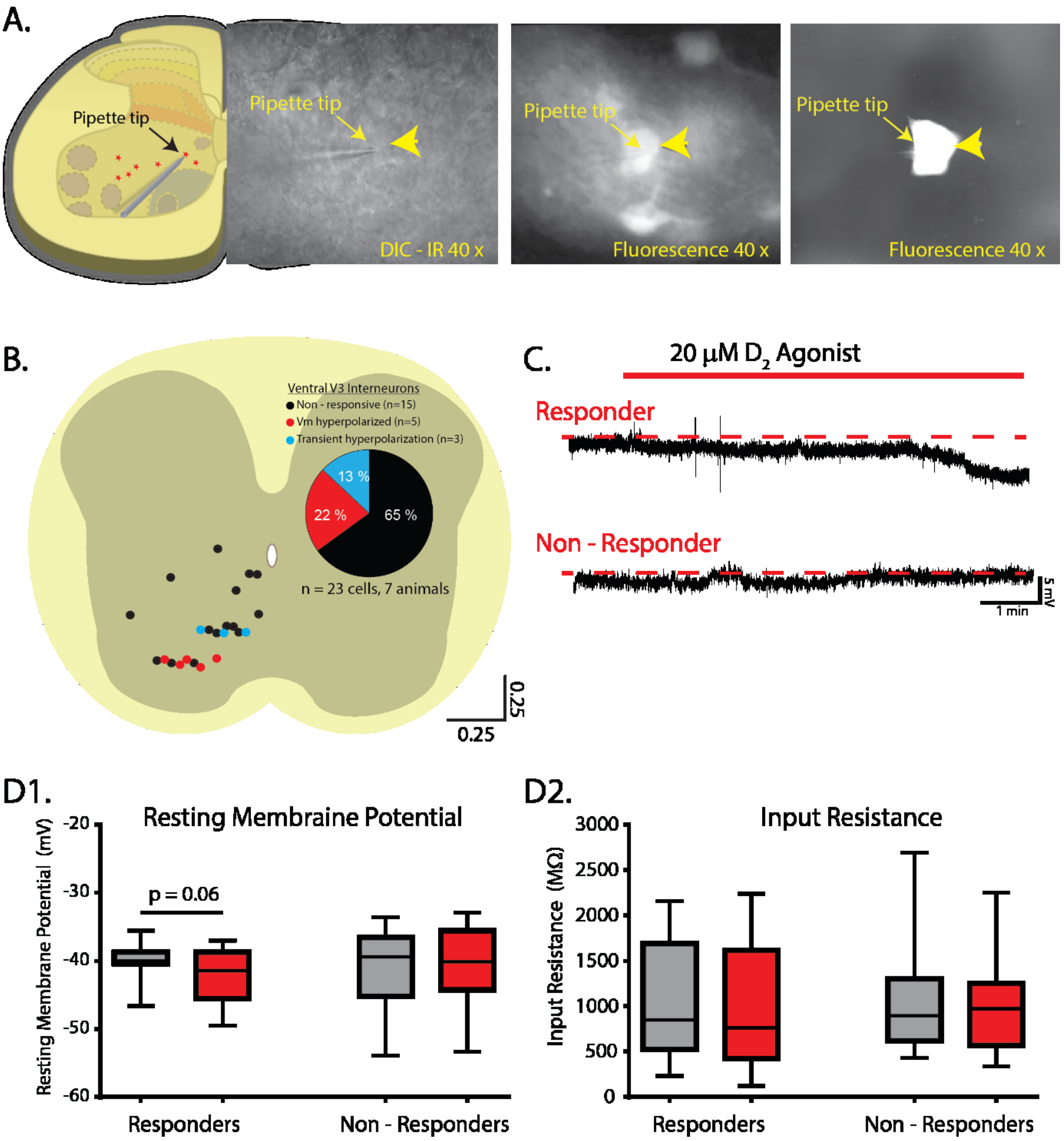

Supplemental Figure 1*: *D2 - receptor control of V3 interneurons.
Whole-cell patch-clamp recordings obtained from lamina V3 interneurons. A. Visually guided patch showing pipette tip (small right-pointing yellow arrows) on a V3 interneuron in DIC-IR, expressing fluorescence for Td-Tomato and filled with a fluorescent dye (large left-pointing yellow arrows). B. V3 interneuron location was measured relative to the central canal and X-Y positions normalized to the distance of ventral and lateral borders of lumbar slices. Non-responders represented by black dots, responders by red dots, and transient responders by blue dots. C. A sample trace showing a hyperpolarization of the resting membrane potential following administration of 20 µM quinpirole in responders (top trace) and non-responders (bottom trace). D1. Box and whisker plots showing a change in resting membrane potential in responders and non-responders before (gray) and after (red) administration of quinpirole. D2. Input resistance graphed in a similar manner as D1.

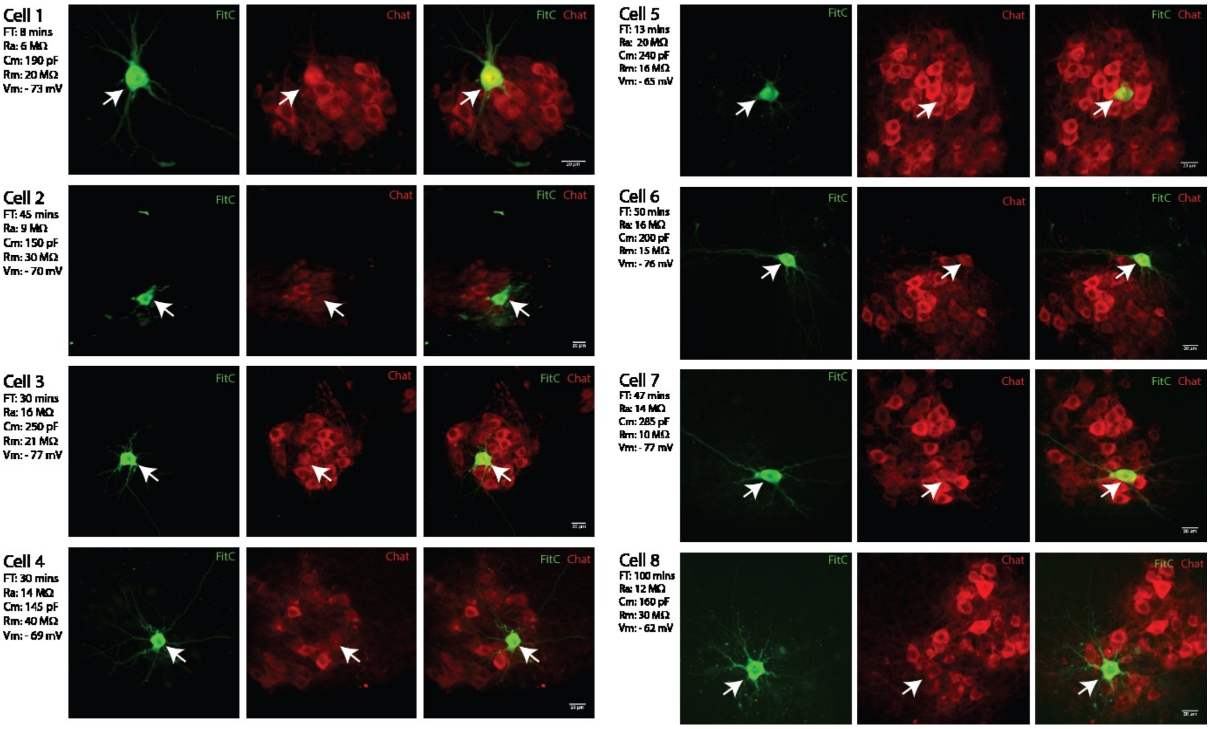

Supplemental Figure 2: Verification of motoneuron identity

Large cells (> 20 um) located in the ventrolateral spinal cord were targeted as putative motoneurons. A cohort of putative motoneurons (n=8) were passively filled with a fluorescent dye, fluorescein dextran amine (FITC - green) added to the intracellular pipette solution and the confirmation of the identity of the filled cells was achieved via *post hoc* labelling for choline acetyltransferase (ChAT - Red), allowing the validation of our approach for identifying motoneurons. Fill times (FT), capacitance (Cm), input resistance (Rin) and resting membrane potential (Vm) are displayed for each cell and were compared with those obtained from recordings in subsequent experiments in Table 1.

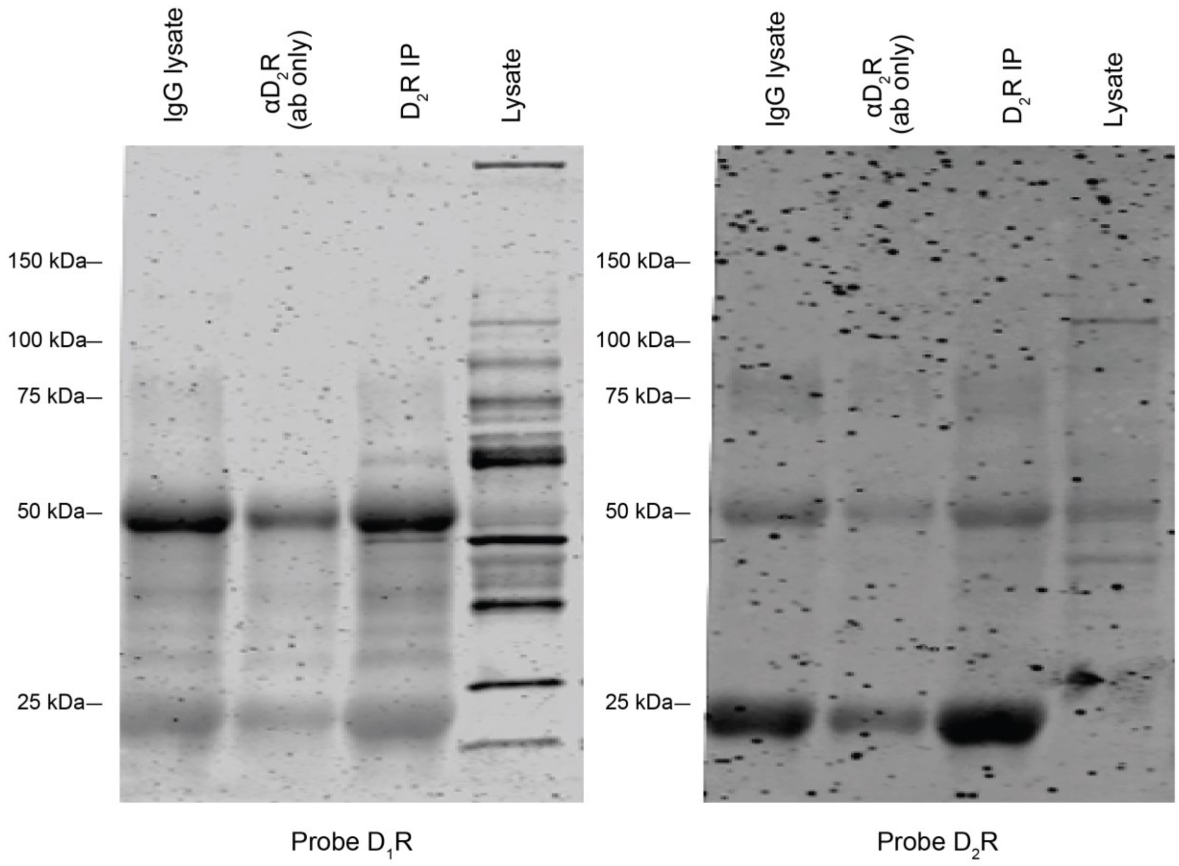

Supplemental Figure 3*: *D2 receptor immunoprecipitation full length blots.
D2 receptors (D2R) were immunoprecipitated from neonatal mouse spinal cord lysates. The IP fraction was run alongside whole spinal cord lysate and antibody controls that include buffer + D2R antibody (ab) and lysate + immunoglobulin antibody (IgG). The blot was probed with D1R antibody (left) and D2R antibody (right).
